# Differences in Hearing Acuity among “Normal-Hearing” Young Adults Modulate the Neural Basis for Speech Comprehension

**DOI:** 10.1523/ENEURO.0263-17.2018

**Published:** 2018-06-08

**Authors:** Yune S. Lee, Arthur Wingfield, Nam-Eun Min, Ethan Kotloff, Murray Grossman, Jonathan E. Peelle

**Affiliations:** 1 Department of Speech and Hearing Science and Chronic Brain Injury, the Ohio State University, Columbus, OH; 2 Volen National Center for Complex Systems, Brandeis University, Waltham, MA; 3 Department of Neurology and Penn Frontotemporal Degeneration Center, University of Pennsylvania, Philadelphia, PA; 4 Department of Otolaryngology, Washington University in St. Louis, St. Louis, MO

**Keywords:** Compensation, fMRI, Hearing, Language, Speech, Young Adults

## Abstract

In this paper, we investigate how subtle differences in hearing acuity affect the neural systems supporting speech processing in young adults. Auditory sentence comprehension requires perceiving a complex acoustic signal and performing linguistic operations to extract the correct meaning. We used functional MRI to monitor human brain activity while adults aged 18–41 years listened to spoken sentences. The sentences varied in their level of syntactic processing demands, containing either a subject-relative or object-relative center-embedded clause. All participants self-reported normal hearing, confirmed by audiometric testing, with some variation within a clinically normal range. We found that participants showed activity related to sentence processing in a left-lateralized frontotemporal network. Although accuracy was generally high, participants still made some errors, which were associated with increased activity in bilateral cingulo-opercular and frontoparietal attention networks. A whole-brain regression analysis revealed that activity in a right anterior middle frontal gyrus (aMFG) component of the frontoparietal attention network was related to individual differences in hearing acuity, such that listeners with poorer hearing showed greater recruitment of this region when successfully understanding a sentence. The activity in right aMFGs for listeners with poor hearing did not differ as a function of sentence type, suggesting a general mechanism that is independent of linguistic processing demands. Our results suggest that even modest variations in hearing ability impact the systems supporting auditory speech comprehension, and that auditory sentence comprehension entails the coordination of a left perisylvian network that is sensitive to linguistic variation with an executive attention network that responds to acoustic challenge.

## Significance Statement

Hearing loss is associated with increased cognitive demand during speech comprehension. Here we used fMRI to measure brain activity while healthy adults with self-reported normal hearing listened to spoken sentences. We found that regions of right frontal cortex, outside of the traditional perisylvian language network, are more active for listeners with poorer hearing as measured with pure-tone audiometry. These findings suggest that executive attention varies with hearing ability, even in the absence of clinical hearing loss, during successful auditory sentence comprehension.

## Introduction

Hearing ability varies considerably from person to person. Although individual differences in auditory sensitivity are most apparent as we age (Cruickshanks et al., 1998; Goman et al., 2017), variability also exists among young adults, including those who would be classified as having clinically normal hearing. The need to investigate the consequences of even mild decrements in hearing acuity is heightened by the increasing use of personal music players and levels of music amplification in concerts and clubs that can approach, and often exceed, potentially dangerous sound levels (Meyer-Bisch, 1996).

It is noteworthy that young adults with hearing impairment are often unaware of it (Le Prell et al., 2011). In part this lack of awareness may be due to upregulation of compensatory neural mechanisms that engage executive or attentional resources in support of central aspects of language processing and compensate for reduced acoustic clarity. Such compensation could result in successful speech understanding despite an attenuated speech signal (Peelle and Wingfield, 2016; Peelle, 2018). While compensatory neural function during speech comprehension has been studied in older adults with hearing impairment (Eckert et al., 2008; Peelle et al., 2011), less attention has been given to the possibility that variability in hearing acuity among young adults with clinically normal hearing may lead them to engage similar operations during spoken language processing.

Studies using functional MRI (fMRI) to investigate speech comprehension generally agree on the importance of bilateral temporal cortex for spoken word processing, with inferior frontal gyrus playing a more important role during sentence comprehension (Hickok and Poeppel, 2007; Rauschecker and Scott, 2009; Peelle et al., 2010a; Adank, 2012; Peelle, 2012). Sentences made more difficult with the use of grammatically complex sentence structures or lengthier sentence materials generally result in further increased left inferior frontal activation, sometimes with the additional activation of homologues in the right hemisphere (Cooke et al., 2002; Peelle et al., 2010b).

Behavioral work with older adults with age-related hearing loss has shown that the additional cognitive processing needed for successful speech recognition can require resources that might otherwise be available for other mental operations (Wingfield et al., 2005). The consequences of this, in the context of limited cognitive resources, can be seen in greater dual-task costs (Tun et al., 2009; Gosselin and Gagné, 2011), poorer episodic memory for what has been heard (McCoy et al., 2005), and decreased comprehension for syntactically complex sentences (Wingfield et al., 2006; DeCaro et al., 2016). The question remains, however, whether hearing acuity affects neural engagement, even among younger adults with clinically normal hearing.

In the present study, we used fMRI to examine the consequences of differences in auditory sensitivity (hearing acuity measured outside of the scanner) for neural engagement during comprehension of spoken sentences in healthy adults with self-reported normal hearing. We hypothesized that additional neural resources would be recruited to compensate for the hearing decrement.

## Materials and Methods

### Subjects

**Figure 1.**
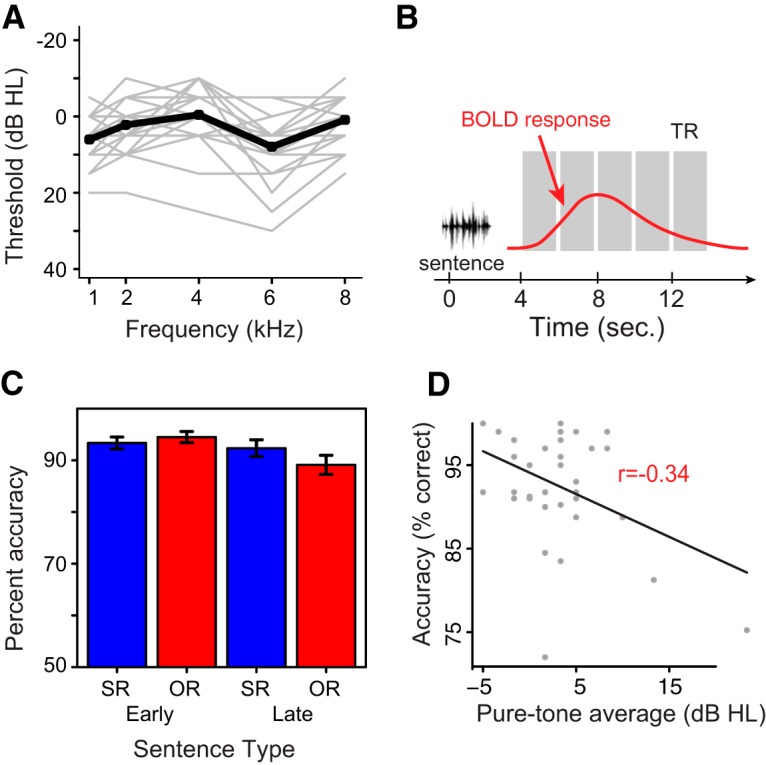
***A***, Pure tone hearing acuity for participants’ better ear in the study. The dark line shows the group average, with individual participant’s hearing levels in light gray. ***B***, Schematic of ISSS sequence used for data collection. Each gray column indicates the window of volume acquisition. ***C***, Accuracy levels for subject-relative (SR) and object-relative (OR) sentences with an adjectival phrase inserted in an early or late sentence position. Error bars indicate one standard error. ***D***, Statistics summary on the behavioral data. Red color indicates significant variables.

Forty-two adults (20 females) from the authors’ University community participated in the study. Participants ranged in age from 18 to 41 years old (mean = 25.8, SD = 4.6). Seven participants were discarded due to poor performance (*n* = 4), excessive head motion (*n* = 1), and lack of working memory data (*n* = 2), leaving us 35 subjects in total. All were self-reported right-handed native speakers of American English, with no known history of neurologic disorders. All participants reported themselves to have normal hearing, although a detailed history (e.g., of noise exposure) was not collected. Participants’ hearing acuity was screened using pure tone audiometry and all fell within a clinically normal range, with a pure tone average (PTA) across 1, 2, and 4 kHz ranging from –5.0 to 23.3 dB HL (mean = 3.5, SD = 5.4; Fig. 1*A*). All participants fell within the range considered clinically normal for speech (PTA <25 dB HL; Katz, 2009). Additionally, participants were administered a working memory task using a reading span test (Daneman and Carpenter, 1980). Instead of the traditional Quasi-Absolute Span Scoring (Daneman and Carpenter, 1980), we have developed a new scoring scheme, Weighed Absolute Span Scoring, in which percentage correct of each set was adjusted by ascending weight (from 1 to 5) to account for difficulty levels. This method also allowed for detecting individual differences between subjects with more variable scores between subjects (mean = 1.76, SD = 0.55). All participants provided written consent as approved by the Human Subjects Institutional Review Board of the authors’ University and were paid for their participation.

### Stimuli

We obtained 24 base sentences consisting of familiar content words from stimuli developed by DeCaro et al. (2016), examples of which are shown in Table 1. Each base sentence included a male or female actor serving equally often as the agent of the action described in the sentence. Indicating the gender of the actor allowed us to monitor participants’ level of comprehension and alertness during the scanning.

**Table 1.** Stimulus categories

Adjective phrase	Position	Sentence example	Correct answer
Subject-relative	Early	Kings with three black horses that *appreciate* queens are good	Male
Subject-relative	Late	Kings that *appreciate* queens with three black horses are good	Male
Object-relative	Early	Kings with three black horses that queens *appreciate* are good	Female
Object-relative	Late	Kings that queens *appreciate* with three black horses are good	Female

* The action verb is italicized and the participant has to indicate gender of the agent performing the action.

We constructed 4 variations for each of the 24 base sentences in a 2 × 2 factorial design by manipulating two sentence-level characteristics, yielding a total of 96 sentences. First, half of the sentences expressed their meaning with a subject-relative center-embedded clause construction, and the other half with a syntactically more complex object-relative center-embedded clause construction. This manipulation was achieved by rearranging word order, thus ensuring that lexical characteristics were matched between subject-relative and object-relative sentences. Second, we inserted an adjectival phrase in either an early or late position in the sentence to explore potential effects of manipulating working memory demands by separating the agent of an action and the action being performed by that agent in the early condition but not the late condition. (As will be indicated, unlike the syntactic manipulation, agency separation had no effect on comprehension accuracy or neural responses for these healthy adults.) Sentences were recorded by a male native speaker of American English.

A subset of 24 sentences was vocoded with a single channel to serve as an unintelligible control condition with similar, speech-like characteristics.

### MRI scanning

Data were collected on a 3 T Siemens Trio scanner (Siemens Medical System) installed with an 8-channel head coil. The field of view was angled ∼30° away from the AC-PC line. Scanning began with acquisition of a T1-weighted structural volume using a magnetization prepared rapid acquisition gradient echo (MPRAGE) protocol [axial orientation, repetition time (TR) = 1620 ms, echo time (TE) = 3 ms, flip angle = 15°, field of view (FOV) = 250 × 188 mm, matrix = 256 × 192 mm, 160 slices, voxel resolution = 0.98 × 0.98 × 1 mm]. Subsequently, 4 runs of blood oxygenation level–dependent (BOLD) functional MRI scanning were performed (TR = 2000 ms, TE = 30 ms, flip angle = 78°, FOV = 192 × 192 mm, matrix = 64 × 64 mm, 32 slices, voxel resolution = 3 × 3 × 3 mm with 0.75-mm gap) using an interleaved silent steady state (ISSS) protocol (Schwarzbauer et al., 2006; Fig. 1*B*). Similar to standard sparse imaging, the ISSS protocol allows us to present auditory stimuli in relative quiet. However, we are able to collect a greater number of images following each stimulus, thereby providing more data per trial while avoiding the main problems of concurrent scanner noise during auditory presentation (Peelle, 2014). In the present study, each ISSS “trial” lasted 14 s: 4 s of relative quiet followed by 10 s (5 volumes × 2 s each) of data collection. We also acquired a 30-direction diffusion-weighted imaging sequence (FOV = 240 mm, matrix size = 128 × 128, number of slices = 70, voxel size = 2 mm isotropic, TR = 8100 ms; TE = 83 ms, fat saturation). Finally, a B_0_ mapping sequence was acquired at the end of the scanning (TR = 1050 ms, TE = 4 ms, flip angle = 60°, FOV = 240 × 240 mm, matrix = 64 × 64 mm, 44 slices, slice thickness = 4 mm, voxel resolution = 3.8 × 3.8 × 4 mm).

### Experimental procedure

Participants underwent 4 fMRI runs, each of which contained both spoken sentences and unintelligible noise (1-channel vocoded speech). Stimuli were presented at a comfortable listening level identified by each participant. Stimuli were presented 1 s after the silent period of the onset of ISSS using MRI-compatible high-fidelity insert earphones (Sensimetrics Model S14). For each sentence, participants were asked to indicate the gender of the character performing the action via button press as quickly and accurately as possible (see Table 1 for examples). For the unintelligible noise stimuli, participants were told to press either of the buttons. Participants held the button box with both hands, using left and right hands for responses (an equal number of male and female responses, paired with left and right hands, were included in all runs). E-Prime 2.0 (Psychology Software Tools) was used to present stimuli and record accuracy.

In each run, there were 24 trials with spoken sentences (2 syntactic types × 2 adjectival phrase positions × 6 sentences each), 6 trials with 1-channel vocoded speech, and 6 trials of silence, totaling 36 trials (8.4 min per run). Sentences were distributed into four runs such that each stimulus associated with a base sentence was in one of the four runs, and the four types of stimulus sentences were equally distributed across the four runs. The order of conditions within each run was randomized. Each of the 96 sentences was presented only once.

Before entering the scanner, participants received instructions and performed a practice session to ensure they understood the task. Once inside the scanner, but before scanning, participants confirmed intelligibility of spoken sentences at the intensity to be used in the main experiment by correctly repeating each sentence as it was presented. These sentences were not included in the main stimulus set. All participants were able to repeat back the sentences accurately, confirming audibility of the stimuli. Once set for a participant, the presentation level did not change during the course of the experiment, although there may have been modest variability in presentation level across participants (see Discussion).

### fMRI data analysis

Preprocessing began by unwarping the functional data using the *prelude* and *flirt* routines from FSL version 5.0.5 (FMRIB Software Library, University of Oxford). The rest of the preprocessing steps were performed using SPM12 (version 6225; Wellcome Trust Center for Neuroimaging): images were realigned to the first image in the series, coregistered to each participant’s structural image, normalized (with preserved voxel size) to MNI space using a transformation matrix generated during tissue class segmentation (Ashburner and Friston, 2005), and spatially smoothed with a 9-mm full-width at half-maximum (FWHM) Gaussian kernel. After preprocessing, the data were modeled using a finite impulse response (FIR) function in which the response in each of the 5 volumes collected with ISSS following an event was separately estimated. We separately modeled trials that yielded correct or incorrect behavioral responses. Additional regressors included 6 motion parameters and 4 run effects. High-pass filtering with a 128-s cutoff was used to remove low-frequency noise. The typical first-order autoregressive modeling for temporal autocorrelation was turned off due to the discontinuous time series.

For all statistical comparisons including *t* tests and regression analyses, we used the integral of the positive portion of the response for the five volumes collected with ISSS, or the summed positive area (SPA; Lee et al., 2016). That is, for each condition, we summed all non-negative parameter estimates and used this single number to reflect the parameter estimate of the neural response during sentence processing. The advantage of using the SPA is that it provides an indication of the overall direction of the effect without relying on assumptions about the shape or latency of the hemodynamic response. Unless specified, all comparisons were done using the SPA associated with correct trials.

All whole-brain results were thresholded using a cluster-forming voxel-wise threshold of *p* < 0.001 (uncorrected) and a cluster-level threshold of *p* < 0.05 (FWE-corrected) across the whole brain based on cluster extent and Gaussian random field theory (Worsley et al., 1992; Friston et al., 1994). Results were projected onto the Conte69 surface-based atlas using Connectome Workbench (http://www.humanconnectome.org/software/connectome-workbench.html) and slices using MRIcron (Rorden and Brett, 2000). Unthresholded statistical maps are available at http://neurovault.org/collections/1950/ (Gorgolewski et al., 2015).

## Results

### Behavioral results

The comprehension accuracy across four different sentence types is shown in Fig. 1*C*. to relate differential performance on the task to hearing acuity, we performed a logistic regression analysis on the trial accuracy data within linear mixed effects (LME) framework in R (version 3.31). In this model, PTA scores, age, working memory, syntactic complexity, adjectival phrase position, and the interaction between PTA score and syntax were specified as fixed effects based on the rationale that these variables have systematic effects on behavioral performance during the sentence comprehension task. Additionally, we estimated variability across subjects by including a random effect in the LME model. Statistical significance was tested within the full LME model. This revealed significant main effects of PTA (*Z* = –2.807, *p* = 0.005) and the interaction between syntactic complexity and PTA (*Z* = 1.974, *p* = 0.048). A detailed summary of all variables is listed in Fig. 1*D*.

Next, given that our behavioral task involved a two-alternative forced choice (2AFC) task, we calculated signal detection theory measures (relative to male as a correct response) to see if there was any behavioral pattern associated with poor hearing ability. To this end, we performed a correlation analysis which revealed that participants with higher PTA thresholds had lower sensitivity (Pearson *r =* –0.38, *p* = 0.02), but there was no relationship between PTA threshold and bias (Pearson *r* = 0.005, *p* = 0.98).

### fMRI results

**Figure 2.**
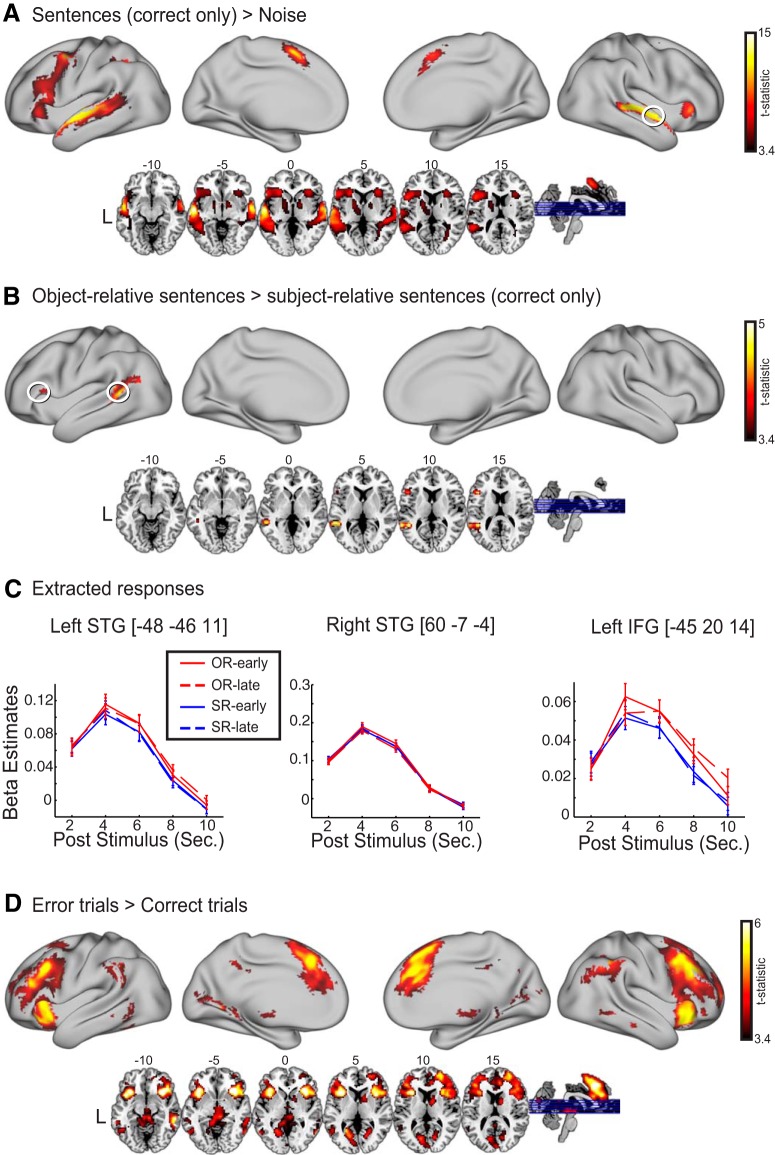
***A***, Increased activity for sentences > noise, limited to sentences with a correct behavioral response. ***B***, Increased activity for object-relative sentences compared to subject-relative sentences, limited to sentences with a correct behavioral response (*p* < 0.053). ***C***, Time course of hemodynamic response from three peak voxels (indicated with white circles). The error bars indicate standard errors. ***D***, Increased activity for trials with errors compared to correct trials.

First, we compared fMRI activity of all sentences to 1-channel noise-vocoded stimuli by performing a paired *t* test using the SPA metric. This comparison yielded a large number of significant clusters in the traditional language regions, including bilateral superior temporal gyrus/sulcus and left inferior frontal gyrus. Other significant regions included left inferior parietal lobule, right cerebellum, and right globus pallidus (Fig. 2*A*; Table 2).

**Table 2.** Maxima of clusters showing a significant effect of auditory sentence processing

Region name	*Z*-score	MNI coordinates, mm			volume of
		*x*	*y*	*z*	cluster, µl
Sentences > noise (correct only)					
Left superior temporal gyrus/sulcus	>10	**–57**	**–10**	**–4**	1,163,700
Left supplementary motor area	7.26	–6	8	56	
Left middle temporal gyrus/sulcus	7.09	–63	–25	2	
Right superior temporal gyrus/sulcus	7.84	**60**	**–7**	**–4**	20,216
Right superior temporal gyrus/sulcus	7.33	66	–13	–1	
Right superior temporal gyrus/sulcus	6.85	60	–25	–1	
Right cerebellum	7.13	**33**	**–64**	**–52**	25,353
Right cerebellum	6.03	27	–61	–28	
Right cerebellum	5.70	36	–67	–25	
Left inferior parietal lobule	6.78	**–27**	**–55**	**41**	8883
Right globus pallidus	4.78	**15**	**–1**	**–1**	6912
Right precentral gyrus	4.14	57	–4	44	
Right putamen	3.76	30	–4	59	
Object-relative > subject-relative					
Left middle temporal gyrus/sulcus	**4.40**	**–48**	**–46**	11	8829
Left middle temporal gyrus/sulcus	4.30	–63	–46	8	
Left middle temporal gyrus/sulcus	4.25	–54	–43	2	

Note: Within each cluster, coordinates of top three maximal loci are listed.

We next compared fMRI activity related to syntactic complexity (object-relative sentences vs. subject-relative sentences) and position of the phrase insertion (early vs. late). We found significant differences in the former comparison in which a significant cluster emerged in the posterior portion of left superior temporal gyrus. An additional cluster, marginally significant in cluster-based correction (*p* = 0.053), was found in left inferior frontal gyrus (Fig. 2*B*; Table 2). By contrast, no significant clusters emerged when early versus late positions of an inserted phrase were compared.

We then extracted time courses in three loci residing in the left superior temporal cortex and left inferior frontal gyrus, as well as a region in right superior temporal cortex for the purpose of reconstructing the hemodynamic response (Fig. 2*C*). As can be seen, these resemble the shape of a canonical hemodynamic response. It can also be seen that the hemodynamic responses in the left hemisphere regions were greater for object-relative sentences than subject-relative sentences, although this difference was not seen in the right hemisphere region.

We also compared error trials to correct trials. This contrast yielded robust error-related activity in a large expanse of bilateral frontal cortex including orbital, dorsolateral, anterior cingulate, inferior frontal, and anterior insula regions. Other regions included angular gyrus, middle temporal gyrus, and posterior cingulate cortex (Fig. 2*D*; Table 3).

**Table 3.** Error-sensitive regions

Region name	*Z*-score	MNI coordinates			Volume of cluster, µl
		*x*	*y*	*z*	
Right middle frontal gyrus	5.55	**48**	**26**	**38**	220,914
Right insula	5.49	36	23	–7	
Right anterior cingulate	5.46	6	32	29	
Right inferior parietal lobule	5.07	**45**	**–52**	**41**	14,229
Right angular gyrus	4.06	54	–61	32	
Right orbitofrontal gyrus	3.65	39	–64	35	
Right middle temporal gyrus	4.88	**54**	**–37**	**–13**	6723
Right middle temporal gyrus	4.83	60	–31	–10	
Right middle temporal gyrus	4.12	57	–61	–1	
Left calcarine sulcus	4.59	**–24**	**–55**	**2**	34,695
Left calcarine sulcus	4.16	–15	–70	8	
Left inferior parietal lobule	4.56	**–48**	**–49**	**41**	21,222
Left supramarginal gyrus	4.08	–60	–49	26	
Left middle temporal gyrus	3.86	–57	–61	–1	
Left precuneus	4.38	**–12**	**–46**	**44**	6264
Left middle cingulate	3.65	–9	–37	38	
Right middle cingulate	3.62	6	–34	38	
Right caudate	3.9	**12**	**2**	**11**	3645
Right pallidum	3.67	12	8	2	

Note: Within each cluster, coordinates of top three maximal loci are listed.

Next, we performed a whole-brain univariate regression in which we separately used PTA, age, working memory, and behavioral accuracy for prediction of the fMRI activity pertaining to correct trials only. This revealed that PTA was the only variable yielding a significant cluster in the right anterior middle frontal gyrus (aMFG). We then performed a series of univariate regressions in which PTA was regressed against each of age, working memory, and behavioral accuracy separately, with the residuals entered into the whole-brain regression. The right aMFG activity still remained significant (Table 4). Lastly, we performed a univariate regression in which PTA was regressed against age, working memory, and behavioral accuracy at once. Although the right aMFG cluster was still observed, this was no longer significant (Table 4). Additionally, we found that this right anterior prefrontal activation was significant for both object-relative sentences and subject-relative sentences when univariate regression with PTA was separately performed with each type of sentence (Table 4). Lastly, we performed the same set of univariate analyses on activity for error trials and unintelligible noise trials; None of these additional analyses yielded significant clusters.

**Table 4.** Maxima of clusters of regression analysis relating hearing level (PTA) to fMRI activity: fMRI results of linear regression analysis

Region name	*Z*-score	MNI coordinates			Volume of cluster, µl
		*x*	*y*	*z*	
PTA					
Right anterior middle frontal gyrus	5.71	30	56	5	3861
PTA (age regressed)					
Right anterior middle frontal gyrus	5.71	30	56	5	3861
PTA (working memory regressed)					
Right anterior middle frontal gyrus	5.23	27	59	5	3429
PTA (accuracy regressed)					
Right anterior middle frontal gyrus	4.61	33	56	5	2862
PTA (age, working memory, accuracy regressed)					
Right anterior middle frontal gyrus	4.14	33	56	5	2214
PTA and SR-correct					
Right anterior middle frontal gyrus	5.58	30	56	5	3402
PTA and OR-correct					
Right anterior middle frontal gyrus	5.51	30	56	2	4077

**Figure 3.**
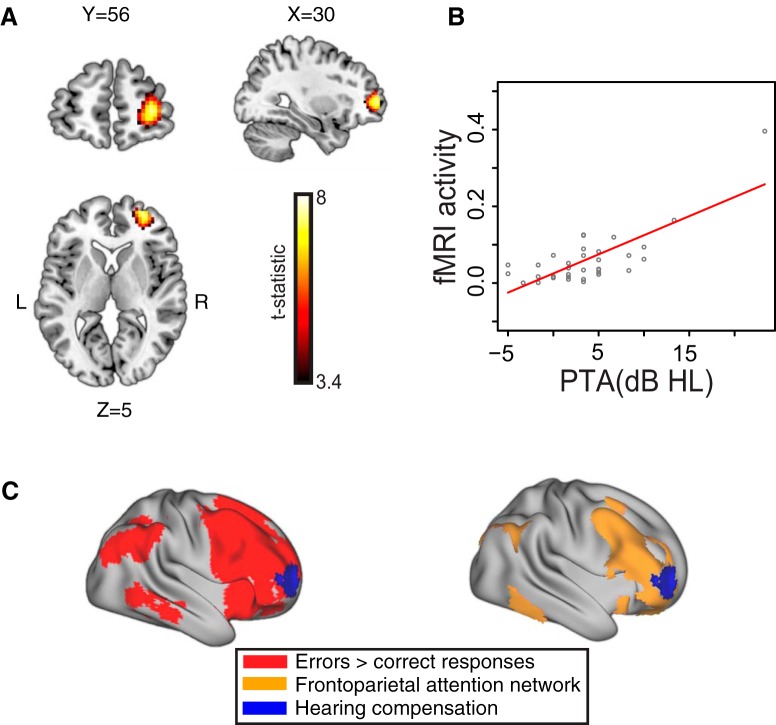
***A***, Right anterior middle frontal gyrus showed greater activity in individuals with poorer hearing acuity in univariate and bivariate regression analyses. The three cross-section views depict the right aMFG cluster from a bivariate analysis where age was regressed from PTA. The *X*, *Y*, and *Z* coordinates (in mm) of the slices are also displayed. ***B***, Correlation between hearing acuity and activity in the right frontal cluster. ***C***, The right frontal cluster overlaid on the error sensitive rendering map (that is, increased activity for error trials relative to correct trials) or the frontoparietal attention network identified using resting state functional connectivity (Yeo et al., 2011).

Fig. 3*B* shows the relationship between PTA and right prefrontal activity. To this end, we averaged SPAs of all voxels in the right prefrontal cluster and related these to PTAs of all subjects. To better characterize the right prefrontal cluster, we compared its location with other contrasts in the current study and known resting state networks (Fig. 3*C*). The right prefrontal cluster overlapped with activation seen in the analysis comparing error trials to correct trials, suggesting that this region participates in monitoring performance in sentence comprehension and incorporates the degree of hearing acuity during auditory sentence processing. It overlaps completely with the frontoparietal attention network defined by Yeo et al., (2011). When the threshold was relaxed (*p* < 0.005, extent cluster size = 30), more clusters were found throughout the frontoparietal attention network, including the right superior frontal gyrus and right posterior STG (not shown; unthresholded image is available from http://neurovault.org/collections/1950/).

## Discussion

During everyday conversation, listeners are generally successful in extracting meaning from speech that varies in its linguistic complexity. Here we have shown that successful sentence comprehension depends in part on the integrated functioning of two networks: the core, left frontotemporal sentence processing network that increased activation depending on syntactic demands; and right anterior middle frontal gyrus, part of the frontoparietal attention network, where activation varied as a function of individual differences in hearing acuity irrespective of syntactic demands. Our data reveal that individual differences in hearing acuity mediate right frontal recruitment even in young adults despite their hearing falling within a range commonly considered to be clinically normal. We discuss the implications of these findings below.

### Linguistic challenge during sentence comprehension

We varied linguistic challenge using subject-relative versus object-relative center-embedded clauses. Comprehending syntactically complex sentences is routinely associated with increased activity in frontotemporal regions, most notably large regions of left frontal cortex and left posterior temporal cortex (Friederici et al., 2003, 2006; Peelle et al., 2004, 2010a; Lee et al., 2016). Here we replicated these findings, with significant increases in left frontal and temporal activity for object-relative sentences compared to subject-relative sentences. This increased activity reflects the additional processing needed to parse the noncanonical word order of the object-relative sentences.

Much prior work has studied the comprehension of grammatically challenging sentences using written materials. The assumption has been that the left perisylvian language network supports a supramodal syntax-processing mechanism (Friederici and Gierhan, 2013). However, auditory presentation inherently involves greater working memory load relative to written presentation because of the transient nature of the stimulus in the auditory modality. While there appear to be some modality-specific differences in sentences presented for reading as opposed to hearing, the differences are generally reflected in increased activation in the association cortices of the presentation modality. Thus, studies of direct comparisons of heard and read sentences generally support the claim that the increased workload associated with increasing grammatical complexity is similar across modalities of sentence presentation (Michael et al., 2001; Constable et al., 2004). Our data indicate that the grammatical manipulation evoked left lateral temporal activation, although it is noteworthy that peak activation was in an anterior-superior temporal distribution within the lateral temporal lobe rather than in a more posterior-inferior distribution also subserving the comprehension of written sentences. Others also have implicated this left anterior-superior temporal region in grammatical processing (Hickok and Poeppel, 2007; Gunawardena et al., 2010).

### Acoustic challenge during sentence comprehension

Although it is useful to think about the core frontotemporal regions involved in understanding intelligible sentences (Scott et al., 2000; Davis and Johnsrude, 2003; Rodd et al., 2005; Rosen et al., 2011; McGettigan et al., 2012), this core network can be modulated by the clarity of the speech signal. For example, altering speech intelligibility using noise vocoding or background noise results in patterns of additional brain activity, most notably increased activity in frontal cortex (Davis and Johnsrude, 2003; Davis et al., 2011; Hervais-Adelman et al., 2012; Wild et al., 2012). Of particular interest is that this modulation can occur when the acoustic clarity of the speech signal is altered but is still entirely intelligible. Our recent work has also shown that completely intelligible noise vocoded sentences yield significant decreases in areas of frontal and temporal cortex, likely due to simplification of the available acoustic information (Lee et al., 2016).

Along with these observations comes mounting behavioral evidence that changing the acoustic clarity of the speech signal has cognitive consequences (Rönnberg et al., 2013; Pichora-fuller et al., 2016; Peelle, 2018). For example, when speech is acoustically degraded, episodic memory is poorer for single words (Rabbitt, 1968; Cousins et al., 2014), word pairs (Heinrich et al., 2008), and short stories (Ward et al., 2016). Poorer hearing and background noise are also associated with greater pupil dilation during listening (Kramer et al., 1997; Kuchinsky et al., 2013; Zekveld and Kramer, 2014; Ayasse et al., 2017), which reflects increased cognitive effort. These findings are consistent with an account in which the processing difficulty of a degraded acoustic signal requires the listener to engage cognitive systems to understand speech, leaving fewer resources available for other operations, such as remembering what has been heard or processing the meaning of sentences with complex syntax (Wingfield et al., 2015). In the present study, we found a mild decrease in auditory sentence comprehension accuracy in individuals with a subtle decrement in PTA.

Although past research has illustrated cognitive consequences of hearing loss in older adults, in the present study we found that differences in hearing acuity in normal-hearing adults can have a significant impact on the brain activation associated with sentence comprehension. That is, the right anterior middle frontal gyrus’s activity was greater in participants whose hearing acuity was poorer. This is particularly intriguing in that the hearing acuity was measured outside of the scanner, and the sound intensity of sentence stimuli was adjusted to the comfortable level during scanning for each individual. This suggests that internal hearing challenge weighs more than mere adjustment of volume to the comfort level with regard to speech comprehension. Importantly, this relationship held true during correct trials, but not error trials, indicating that upregulation of right aMFG may lead to successful speech comprehension for those who have mild hearing decrement despite still falling within a range defined as clinically normal hearing for speech (Katz, 2009).

### Executive attention systems for error control and online resource allocation

One of our main interests was to investigate the degree to which brain regions outside the core speech network are recruited to facilitate comprehension. Two particularly relevant systems are the cingulo-opercular and frontoparietal executive attention networks (Power and Petersen, 2013). In our current data, we observed robust activity in both networks in response to error trials. Prior literature, however, demonstrates that these two attention networks serve dissociable roles in support of task performance (Neta et al., 2015). Importantly, for correct responses, we observed activity in right anterior middle frontal gyrus, part of the frontoparietal attention network. This activation was significantly correlated with hearing acuity, such that listeners with poorer hearing showed greater activity than those with better hearing. Our findings are consistent with the dissociation of these frontal-mediated executive attention systems in that we only saw activation in the frontoparietal executive attention network in response to sentence comprehension with the perceptual challenge of reduced hearing acuity.

The cingulo-opercular network includes the dorsal anterior cingulate and bilateral frontal operculum (and/or anterior insula). Consistent with its role in error monitoring, activity in the cingulo-opercular network is frequently seen in speech comprehension tasks under conditions where intelligibility is reduced. This includes single words in noise (Eckert et al., 2009; Vaden et al., 2015) and noise-vocoded sentences (Wild et al., 2012; Erb et al., 2013). Critically, the level of cingulo-opercular activity following a perceptual error relates to success on the following trial (Vaden et al., 2013) and memory for what has been heard (Vaden et al., 2017), suggesting that cingulo-opercular activity plays a causal role in successful speech perception, perhaps by re-engaging listeners in the current task set.

By contrast, the frontoparietal attention network includes bilateral inferior parietal cortex and dorsolateral prefrontal cortex, and is frequently implicated in flexible application of task demands (Duncan, 2010; Hampshire et al., 2011; Woolgar et al., 2011; Stokes et al., 2013), particularly in the context of working memory tasks (Owen et al., 2005). What was most notable in the current data were the selective engagement of a subset of the frontoparietal network—right anterior middle frontal gyrus—as a function of individuals’ hearing acuity, even among listeners who self-reported normal hearing and whose audiograms would be considered clinically normal. Located in right anterior middle frontal gyrus, this region is distinct from the cingulo-opercular network. Thus, both its anatomic location (overlapping the frontoparietal attention network) and response characteristics (engagement in intelligible sentence trials, not responsive to unintelligible noise trials depending on hearing acuity) suggest that this is distinct from the cingulo-opercular network. Moreover, this region is not part of the core sentence-processing network: right middle frontal activation was independent of syntactic demands, showing correlations with hearing acuity regardless of syntactic construction.

### Other considerations

In the present study, we employed an advanced auditory fMRI protocol, ISSS, which allowed multiple time points of data acquisition following the presentation of a stimulus during a silent period. From a data-analytic perspective, this posed a unique challenge such that the conventional modeling scheme (i.e., convolving with a canonical HRF) is not straightforward. As described in Methods, we used an FIR model as an initial step to estimate an unbiased response at each time point. Then, by taking the integral of all beta estimates across consecutive time points, we computed a summed positive response area (SPA). Although this approach may be less sensitive than FIR (Perrachione and Ghosh, 2013), one advantage of SPA over FIR is that it provides a single, temporally unbiased measure of response for each condition for each participant, facilitating group-level statistical analysis. In a previous study (Lee et al., 2016) we attempted numerous analytic approaches to similar ISSS data, including using a canonical HRF (with and without derivatives), FIR, and the SPA approach described here. Empirically we observed the clearest “expected” patterns for sentence processing with the SPA approach. However, we acknowledge that this warrants more exploration in the future studies.

Second, the sound presentation level for each participant was chosen after a few rounds of adjusting the intensity that was most comfortable before the experiment. Once set, the volume was fixed over the course of the study. We adjusted the presentation volume for each participant to ensure comfort and audibility but acknowledge that this approach potentially changed the stimulation level across participants. However, we note that this did not work against our hypothesis and finding, in that PTAs still predicted both behavioral performance and activity in right aMFG. Last, despite finding that right aMFG was the only significant region across univariate and bivariate regression analyses predicted by PTA, we acknowledge that the right aMFG cluster did not survive significance when the PTA metric was regressed against all variables taken together, including age, working memory, and accuracy. This is presumably due to the relatively small sample size for the whole-brain regression analyses. Nonetheless, our several confirmatory analyses involving error and noise trials and significant correlation result of PTA and right aMFG activity without outliers suggest that right aMFG plays a role in compensating for modest hearing decrement in young adults.

### Conclusions

Although noise-induced hearing loss is often thought of in terms of exposure to explosive blast, power tools, recreational vehicles, or unprotected industrial noise (Fligor, 2009), risks to hearing from personal music players at maximum volume and heavily amplified music in entertainment venues has been an increasing cause for concern (Meyer-bisch, 1996), and many university-aged young adults may have developed a mild decrement in hearing acuity without awareness (Widén et al., 2009; Le Prell et al., 2011). It is now known that there are consequences of hearing impairment beyond simply missing or mishearing words in everyday conversations and the voice of a university lecturer: even when spoken words are correctly perceived, the effort needed to attain this success may come at the cost of cognitive resources that would otherwise be available for encoding what has been heard in memory or comprehension of linguistically demanding complex speech. One might ordinarily assume that adults who may have a mild decrement in hearing—but are still within a clinically normal range—would be immune from such effects. In this report, we show that, counter to such an assumption, subtle variations in hearing sensitivity can indeed affect accuracy of sentence comprehension and increase neural engagement for comprehension success of a nonlinguistic component of the neural network recruited to support sentence comprehension.

## Synthesis

Reviewing Editor: Morgan Barense, University of Toronto Decisions are customarily a result of the Reviewing Editor and the peer reviewers coming together and discussing their recommendations until a consensus is reached. When revisions are invited, a fact-based synthesis statement explaining their decision and outlining what is needed to prepare a revision will be listed below. The following reviewer(s) agreed to reveal their identity: Kenneth Vaden, Stefanie Kuchinsky

There was general agreement that the revised manuscript is much improved, and that the paper raises interesting questions about the impact of elevated hearing thresholds on performance and brain activity, despite having hearing with the normal range. We all believe it to be an important contribution to the literature.

There are only two remaining questions/comments:

1) Page 10. Do other PTA clusters appear within the FPN-distribution at a lower voxel statistic threshold? If so, this might be worth noting to help support that aMFG responded as part of the broader FPN.

2) The scatterplot showing aMFG activity in relation to PTAs is fine as an illustration of a significant cluster-level effect (Fig 3B). However, it is not kosher to report the correlation and its significance (page 10, lines 8-9; Fig 3B) given that the cluster was identified using significant PTA correlations (Vul et al., 2009). The extreme value can be noted as having a non-critical contribution to the effect without performing the circular correlation. Likewise, it is sufficient to report that no cluster-level age effects were observed in whole-brain statistic tests so Fig 3B does not need the age scatterplot. Since the whole brain statistic results indicate clusters that are PTA-related and not age-related, reporting those results should be sufficient.

Vul, E., Harris, C., Winkielman, P., & Pashler, H. (2009). Puzzlingly high correlations in fMRI studies of emotion, personality, and social cognition. Perspectives on psychological science, 4(3), 274-290.

Minor

1. Some issues with text displayed in Figure 3B. The X-axis of the top subplot in 3B and the green text in the bottom subplot of 3B are displayed as nonsense characters.
